# miR-101 sensitizes human nasopharyngeal carcinoma cells to radiation by targeting stathmin 1

**DOI:** 10.3892/mmr.2015.3221

**Published:** 2015-01-19

**Authors:** QUANQUAN SUN, TONGXIN LIU, TIAN ZHANG, SHASHA DU, GUOZHU XIE, XIAOSHAN LIN, LONGHUA CHEN, YAWEI YUAN

**Affiliations:** Department of Radiation Oncology, Nanfang Hospital, Southern Medical University, Guangzhou, Guangdong 510515, P.R. China

**Keywords:** microRNA, nasopharyngeal carcinoma, radioresistance, miR-101, stathmin 1

## Abstract

Radioresistance remains a major problem in the treatment of patients suffering from nasopharyngeal carcinoma (NPC). A better understanding of the mechanisms involved in the induction of radioresistance may provide strategies to improve NPC patients’ response to therapy. The present study aimed to investigate the effect of microRNA (miR)-101 on the radioresistance of NPC cells. Analysis of miR-101 expression levels indicated that miR-101 was downregulated in NPC cell lines. Furthermore, ectopic expression of miR-101 suppressed cell proliferation and enhanced radiosensitivity of NPC cells. Stathmin 1 (STMN1) was additionally verified as a direct functional target of miR-101, which was found to be involved in cell viability, radioresistance and radiation-induced autophagy of NPC cells. In conclusions, the results of the present study suggested that the identified miR-101/STMN1 pathway contributed to the elucidation of the mechanisms of radioresistance in human NPC and that it may represent a potential therapeutic target.

## Introduction

Nasopharyngeal carcinoma (NPC) is one of the most common types of cancer derived from epithelial cells located in the nasopharynx (1). NPC has a distinct epidemiology and distribution, with the highest incidence in southern China and Southeast Asia (2). Radiotherapy is the primary therapeutic strategy for patients with NPC (3). Although numerous patients with NPC undergo radiotherapy treatment, advanced NPC patients tend to experience therapy failure due to local recurrence and distant metastasis (4). The poor prognosis of NPC patients may be due to its radioresistance (5). An improved understanding of the molecular mechanisms that contribute to the radioresistance of NPC may provide novel therapeutic strategies and therefore improved clinical outcomes.

microRNAs (miRNAs) are a class of small (19–25 nucleotides) endogenous non-coding RNA molecules that regulate gene expression at the post-transcriptional level by translational arrest or messenger RNA (mRNA) cleavage. These effects are achieved via miRNA-binding to the 3′-untranslated region (3′UTR) of their target mRNA, which decreases expression of the associated protein (6). An increasing body of evidence suggests that miRNAs have crucial roles in numerous biological processes, including cellular differentiation, proliferation, apoptosis, autophagy and metabolism (7,8). The dysregulation of miRNAs may be associated with numerous human diseases, including certain types of cancer (9). Specific miRNAs function as tumor suppressors or oncogenes and are influential in tumor progression and therapeutic resistance (10). Of note, recent studies have indicated an association between the expression of certain miRNAs, including miR-608 and miR-29c, and the success of radiotherapy treatment, particularly in NPC. (11,12). Further studies have indicated that miR-101 is downregulated in numerous types of cancer, including gastric (13), lung (14) and colon cancer (15), and a loss of miR-101 expression is involved in carcinogenesis (16) and angiogenesis (17). Studies have also indicated that the ectopic expression of miR-101 is able to sensitize non-small cell lung cancer cells to radiation by targeting DNA-dependent protein kinase and ataxia telangiectasia mutated (ATM) (18). However, the association between miR-101 and the modulation of radioresistance in NPC remains to be elucidated.

The present study aimed to elucidate the function of miR-101 in NPC by analyzing miR-101 expression in NPC cell lines and investigating the effects of ectopic expression on NPC-cell proliferation and radiosensitivity. Additionally, the present study aimed to identify functional targets of miR-101 in order to elucidate the mechanism by which it exerts its effects in NPC and thereby propose a strategy for enhancing NPC cell radiosensitivity and improving treatment of NPC.

## Materials and methods

### Cell culture

Three NPC cell lines (CNE-2, 5-8F and 6-10B) were provided by Prof. Xia Yunfei (Sun Yat-sen University Cancer Center, Guangzhou, China) and maintained in the State Key Laboratory of Oncology in South China (Guangzhou, China). The CNE-1 and NP69 cell lines were maintained in the lab at Nanfang Hospital, Southern Medical University (Guangzhou, China) and were purchased from the Cell Bank of Sun Yat-Sen University in 2010. The passage number of all five cell lines used in the present study was <20. All of the cell lines were tested against mycoplasmic infection. CNE-1, CNE-2, 5-8F and 6-10B cells were maintained in RPMI-1640 medium (Invitrogen Life Technologies, Carlsbad, CA, USA) supplemented with 10% fetal bovine serum (FBS; Invitrogen Life Technologies), 100 U/ml penicillin (Sigma-Aldrich, St. Louis, MO, USA) and 50 μg/ml streptomycin (Sigma-Aldrich). NP69 cells were cultured in keratinocyte/serum-free medium (Invitrogen Life Technologies) supplemented with bovine pituitary extract (BD Biosciences, San Jose, CA, USA). All cell lines were incubated at 37°C in a humidified atmosphere of 5% CO_2_.

### Quantitative polymerase chain reaction (qPCR)

Total RNA was isolated from cells using Trizol reagent (Invitrogen) according to the manufacturer’s instructions. The reverse-transcription and PCR primers for miR-101 and U6 were purchased from Ribobio (Guangzhou, China). The PCR primers for STMN1 were forward, 5′-CCTCTGTTTGGCGCTTTTGTGCG-3′ and reverse, 5′-GGCACGCTTCTCCAGTTCTTTCACC-3′. The PCR primers for β-actin were forward, 5′-TCGACAACGGCTCCGGCAT-3′ and reverse, 5′-AAGGTGTGGTGCCAGATTTTC-3′. The cDNA library was synthesized using the PrimeScript RT reagent kit (Takara Bio, Inc., Dalian, China). For mature miRNA quantification, cDNA was generated using specific stem-loop universal primers. Aliquots of cDNA were amplified for 40 cycles, which were perofmred as follows: Denaturing at 95°C for 5 sec, annealing at 60°C for 34 sec and extension at 60°C for 1 min. Real-time qPCR for miRNA and mRNA was performed using SYBR^®^ Premix Ex Taq II (Takara) and quantified in an ABI 7500 Sequence Detection system (Perkin Elmer/Applied Biosystems). Either U6 or β-actin were used as an internal control.

### Oligonucleotide and small interfering RNA (siRNA) transfection

miR-101 mimic, miRNA mimic negative control oligonucleotides, STMN1 siRNA and siRNA negative control were all purchased from Ribobio. Oligonucleotide and siRNA transfection were performed using Lipofectamine^®^ 2000 reagent (Invitrogen) according to the manufacturer’s instructions.

### Cell proliferation assay

Cell proliferation was measured using the MTT dye reduction method (19). Briefly, 48 h following transfection, cells were seeded in 96-well plates at a low density (2×10^3^ cells/well) in RMPI-1640 medium supplemented with 10% FBS and incubated for 1–5 days. Following incubation, 50 μl MTT solution (2 mg/ml; Sigma-Aldrich) was added to each well, and the cells were incubated for a further 2 h at 37°C. The media containing MTT solution was removed, and the dark blue crystals were dissolved by the addition of 100 μl dimethylsulfoxide. The absorbance value was measured with a microplate reader (SpectraMax M5; Molecular Devices, Sunnyvale, CA, USA) at a test and reference wavelength of 570 nm. The percentage of growth was determined relative to untreated controls. Each experiment was performed ≥three times with triplicate samples.

### Clonogenic survival assays

CNE-2 and 5-8F cells were pretreated by either miR-101 mimic or STMN1 siRNA transfection for 48 h and subsequently seeded onto six-well plates in triplicate at specific cell densities, followed by exposure to the indicated doses of radiation (0, 2, 4, 6 or 8 Gy) using 6 MV X-rays generated from linear accelerators (Varian 2300EX; Varian, Palo Alto, CA, USA) at a dose rate of 3 Gy/min. Following 10–14 days of incubation at 37°C, the cells were fixed using 100% methanol and stained using 1% crystal violet (Sigma-Aldrich). Colonies containing ≥50 normal-appearing cells were counted via microscopic inspection (Olympus IX71; Olympus, Tokyo, Japan). The surviving fraction was calculated as described previously (20). The multi-target single-hit model was fitted to the data to generate survival curves using the formula: SF=1−(1−e^−D/D0^)^N^. The sensitization enhancement ratio at a survival fraction of 10% (SER10) was subsequently calculated. Each experiment was independently performed ≥three times.

### Immunofluorescent staining for γ-H2AX

Forty-eight hours following transfection with miR-101 mimic or miRNA mimic negative control, 1×10^5^ cells were seeded in chamber slides and incubated overnight. The cells were subsequently exposed to 6 Gy irradiation (IR). Twenty-four hours following IR, the cells were fixed in 4% paraformaldehyde (Sigma-Aldrich), permeabilized in 0.1% Triton X-100 (Sigma), blocked in 2% bovine serum albumin (Roche, Stockholm, Sweden) and incubated with a primary antibody against γ-H2AX (Abcam, San Francisco, CA, USA) overnight at 4°C. The primary antibody was subsequently washed off, and a secondary antibody conjugated to fluorescein isothiocyanate (Santa Cruz Biotechnology, Inc., Santa Cruz, CA, USA) was applied to the slides. Cells were washed with phosphate-buffered saline (Sigma-Aldrich) and counterstained with DAPI (Invitrogen Life Technologies). The γ-H2AX foci were observed under a fluorescence microscope (Olympus BX51, Olympus). For each group, the γ-H2AX foci were counted in ≥50 cells.

### Antibodies and western blot analysis

For the western blot analysis, cells were lysed in radio-immunoprecipitation assay buffer (Cell Signaling Technology, Beverly, MA, USA) containing phosphatase and proteinase inhibitor cocktails (Sigma-Aldrich). The protein concentrations were determined using a bicinchoninic acid protein assay kit (Pierce Biotechnology, Rockfold, IL, USA). Equal amounts of total protein were resolved via SDS-PAGE (Bio-Rad, Hercules, CA, USA), and the proteins were transferred onto polyvinylidene difluoride membranes (Bio-Rad). The membranes were blocked in 5% non-fat milk for 1 h at room temperature and then incubated overnight at 4°C with primary antibodies to anti-STMN1 (Abcam), anti-LC3B (Novus Biological, Littleton, CO, USA), anti-p62/SQSTM1 (Cell Signaling Technology) or anti-β-actin (ProteinTech, Chicago, IL, USA). Following three washes, the membranes were incubated with secondary antibodies [species-specific horseradish peroxidase(HRP)-conjugated] for 1 h at room temperature. The immunoreactive bands were visualized with the Immobilon Western chemiluminescent HRP substrate (Millipore, Temecula, CA, USA). Each experiment was independently performed ≥three times.

### Generation of stable green fluorescent protein (GFP)-light chain (LC)3-expressing cells

The lentiviral vector containing the GFP-LC3 reporter was purchased from GenePharma (Shanghai, China). CNE-2 cells were infected with recombinant lentivirus and purified using flow cytometry (BD Biosciences) to generate populations that stably expressed GFP-LC3.

### Luciferase reporter assay

The STMN1 wild-type (wt) and mutant (mut) 3′UTRs, which contain the putative miR-101 binding site, were created and cloned into the Renilla luciferase vector (pLUC-REPORT vector; Promega, Madison, WI, USA). For the luciferase reporter assay, CNE-2 and 5-8F cells were co-transfected with a luciferase reporter vector (either pLUC-3′UTR-STMN1 or pLUC-3′UTR-mut-STMN1) and negative control miRNA or the miR-101 mimic. Forty-eight hours following transfection, the cells were assayed for luciferase activity using the Dual-Luciferase assay kit (Promega) according to the manufacturer’s instructions. For each sample, the relative luciferase activity was normalized to firefly luciferase activity. Three independent experiments were performed in triplicate.

### STMN1 rescue experiments

The pCMX-IRES2-eGFP-STMN1 (PCA-STMN1) plasmid and empty vector were synthesized by GenePharma (Shanghai, China). CNE-2 cells were co-transfected with miR-101 mimic or miRNA mimic negative control and with pCMX-IRES2-eGFP-STMN1 plasmid or the empty vector. Forty-eight hours following transfection, the cells were analyzed for proliferation and clonogenic survival as described. The cells were also analyzed for radiation-induced autophagy activity by western blotting and confocal microscopy (Olympus FV1000; Olympus). STMN1 expression was verified by western blot analysis.

### Statistical analysis

All values were expressed as the mean ± standard deviation and were obtained from experiments that were repeated ≥three times. Significant differences between the means were measured using a two-tailed unpaired Student’s t-test or one-way analysis of variance. All statistical analyses were performed using SPPS version 13.0 software (SPSS Inc., Chicago, IL, USA). P<0.05 was considered to indicate a statistically significant difference between values.

## Results

### miR-101 is downregulated in NPC cell lines and affects the radiation response of NPC cells

**Figure 1:**
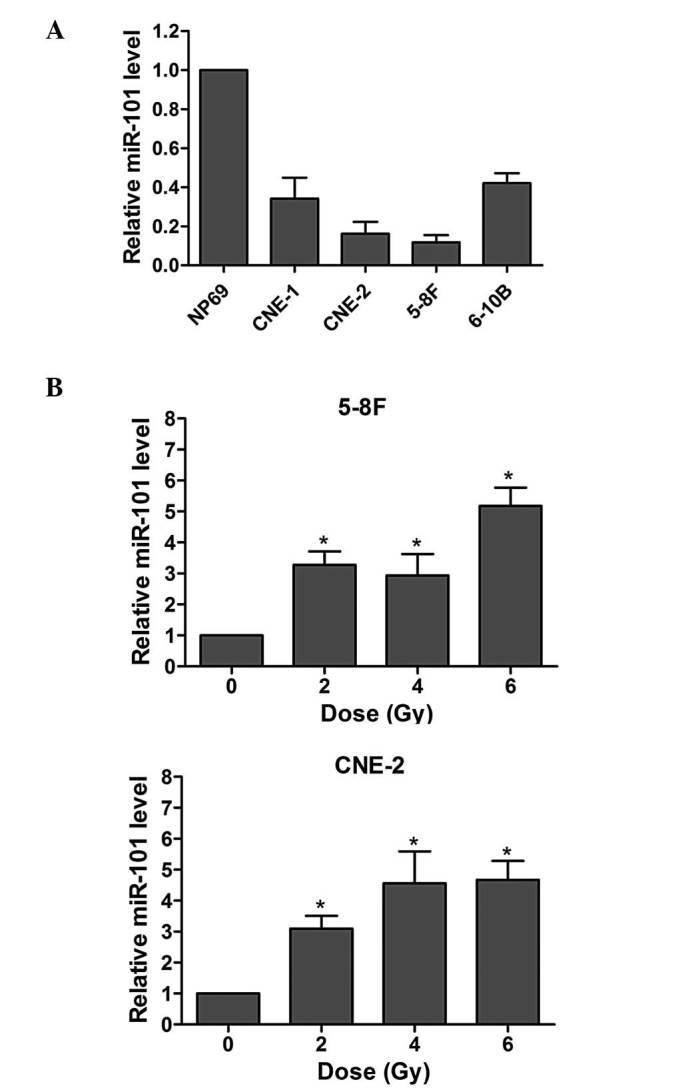
miR-101 is downregulated in NPC cell lines and influences the radiation response of NPC cells. (A) Relative expression of miR-101 in NP69 and NPC cell lines. (B) Levels of IR-induced miR-101 expression in CNE-2 and 5-8F cells increased following various doses of IR for 24 h. U6 was used as the internal control. Values are presented as the mean ± standard deviation (n=3). ^*^P<0.05 vs. 0 Gy. miR, microRNA; NPC, nasopharyngeal carcinoma; IR, irradiation.

In the present study, the expression of miR-101 in four NPC cell lines and the human immortalized nasopharyngeal epithelial cell line NP69 was investigated. As indicated in Fig. 1A, the miR-101 levels were significantly decreased in all four NPC cell lines (P<0.01), particularly in the 5-8F and CNE-2 cell lines.

The effects of radiation on miR-101 expression of NPC cells were also examined. As demonstrated in Fig. 1B, the levels of IR-induced miR-101 expression in both cell lines increased upon IR. These results indicated that miR-101 may influence the IR response of NPC cells.

### miR-101 suppresses NPC-cell viability and sensitizes NPC cells to radiation

**Figure 2:**
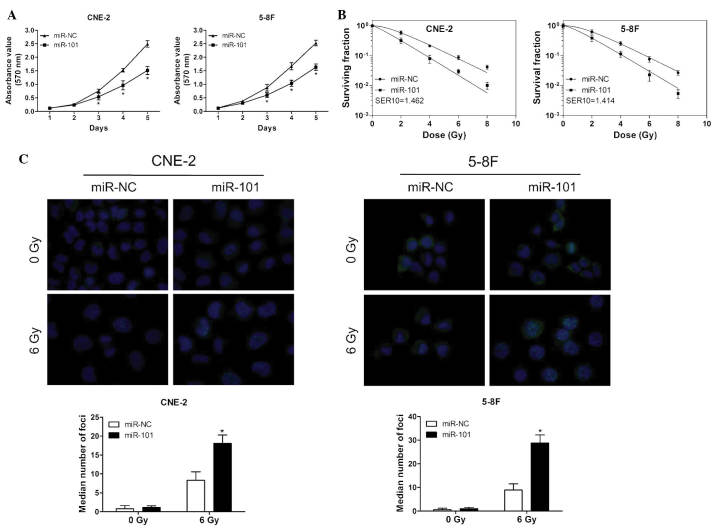
miR-101 suppresses NPC cell viability and sensitizes NPC cells to IR. (A) Cell viability was determined following transfection with miR-101 mimic or miRNA mimic NC at day 1–5 in CNE-2 and 5-8F cells. (B) Clonogenic survival assays of CNE-2 and 5-8F cells treated with miR-101 mimic or miRNA mimic NC followed by various doses of irradiation. Surviving fractions were calculated as described. SER10, sensitizer enhancement ratio at 10% survival. (C) γ-H2AX foci formation was determined in CNE-2 and 5-8F cells transfected with miR-101 mimic or miRNA mimic negative control 24 h following IR. DAPi staining was performed and micrographs were captured at magnification ×400. The median number of foci formation is presented in bar graphs. Values are presented as the median ± standard deviation. ^*^P<0.05 vs. 6 Gy. miR. NPC, nasopharyngeal carcinoma; IR, irradiation; miR, microRNA; NC, negative control.

To evaluate the effects of miR-101 overexpression on the cell viability and radiosensitivity of NPC cells, an MTT assay and a clonogenic survival assay were performed following transfection of CNE-2 and 5-8F cells with miR-101 mimic or negative control. The ectopic expression of miR-101 significantly reduced the proliferation of NPC cells compared with that of the controls (Fig. 2A). Moreover, the survival fraction of cells transfected with miR-101 mimics was significantly decreased following various doses of irradiation compared with that of cells transfected with negative controls (Fig. 2B).

To assess the effect of miR-101 on DNA damage in NPC cells, the number of γ-H2AX foci following IR was measured. The γ-H2AX foci-number is an established molecular marker of DNA damage and repair (21). As indicated in Fig. 2C, the ectopic expression of miR-101 led to a markedly increased persistence of γ-H2AX foci 24 h post-IR compared with that of the control groups. These results suggested that miR-101 enhanced the radiosensitivity of NPC cells in a manner that may be associated with the suppression of cell viability and persistence of DNA damage.

### STMN1 is a direct target of miR-101 and is involved in NPC-cell radioresistance and growth

**Figure 3:**
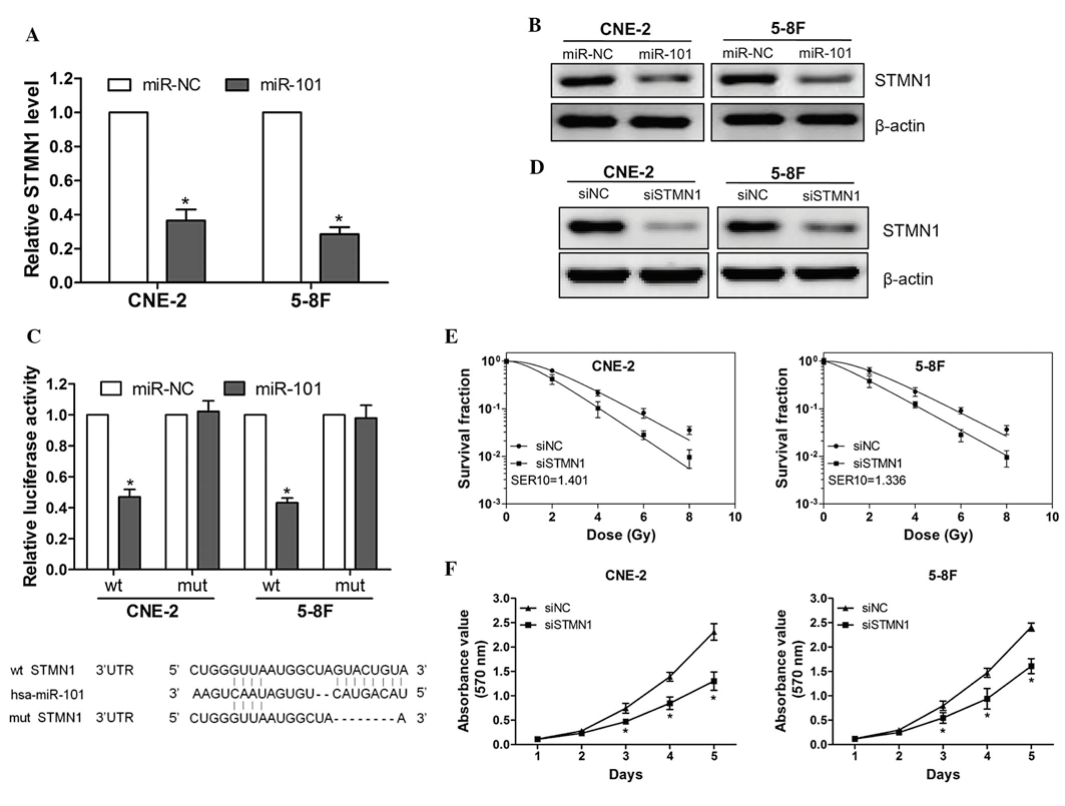
STMN1 is a direct target of miR-101 and involved in NPC cell radioresistance and growth. (A and B) Quantification of (A) STMN1 protein levels and (B) mRNA expression following transfection with miR-101 mimic or miRNA mimic NC. (C) CNE-2 and 5-8F cells were co-transfected with a wt or mut STMN1 3′UTR reporter gene and a miR-101 mimic or a miRNA mimic NC. Wt and mut miR-101 target sequences of the STMN1 3′UTR are indicated. (D) Western blot analysis for STMN1 48 h following transfection with STMN1 siRNA or a negative control. (E) Clonogenic survival assays of CNE-2 and 5-8F cells treated with STMN1 siRNA or an NC followed by various doses of radiation. Surviving fractions were calculated as described. SER10, sensitizer enhancement ratio at 10% survival. (F) Cell viability was determined following transfection with STMN1 siRNA or an NC at days 1–5 in CNE-2 and 5-8F cells. Values are presented as the mean ± standard deviation. ^*^P<0.05 vs miR-NC. STMN1, stathmin 1; miR, miRNA; NC, negative control; wt, wild-type; mut, mutant; siRNA, small interfering RNA; UTR, untranslated region; hsa, *Homo sapiens*.

To investigate the molecular mechanism by which miR-101 increased the radiosensitivity of NPC cells, STMN1 was identified as a potential target of miR-101 based on the three publicly available databases [TargetScan (http://www.targetscan.org), miRanda (http://www.microrna.org/microrna/home.do) and Pictar (http://pictar.mdc-berlin.de/)] and a previous study (7). The ectopic expression of miR-101 was able to significantly suppress the mRNA and protein expression of STMN1 (Fig. 3A and B). Subsequently, luciferase reporter vectors that contained wild-type or mutant miR-101 target sequences of the STMN1 3′UTR were constructed (Fig. 3C, lower panel) and a luciferase reporter assay was performed to determine whether STMN1 was a direct target of miR-101. It was demonstrated that the overexpression of miR-101 significantly suppressed the luciferase activity of the wt 3′UTR of STMN1 but not the mut reporter gene (Fig. 3C, upper panel), indicating the specificity of miR-101 to target the STMN1 3′UTR. These results indicated that STMN1 is a direct target of miR-101 in NPC cells.

To confirm that the miR-101-enhanced radiosensitivity is due to the direct targeting of STMN1, CNE-2 and 5-8F cells were transfected with STMN1 siRNA or control siRNA. Knocking down the expression of STMN1 significantly enhanced the radiosensitivity of CNE-2 and 5-8F cells (Fig. 3D and E). Furthermore, the proliferation assay indicated that silencing STMN1 expression significantly suppressed CNE-2 and 5-8F cell-growth (Fig. 3F). These data demonstrated that miR-101 enhanced radiosensitivity by directly targeting STMN1.

### Restoration of STMN1 expression rescues the effect of miR-101 on cell viability, radiosensitivity and radiation-induced autophagy

**Figure 4:**
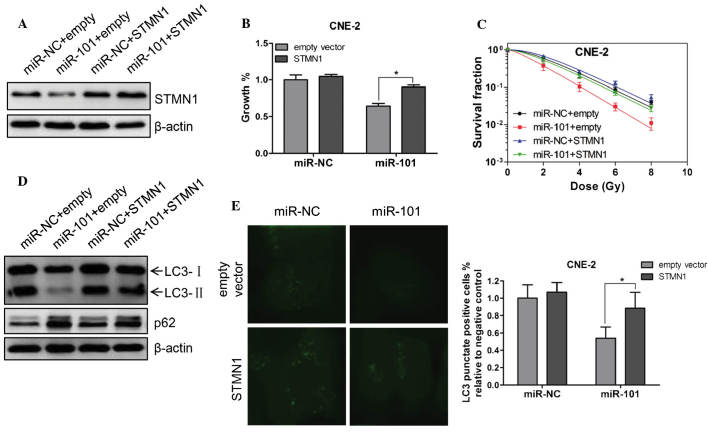
Restoration of STMN1 expression rescues the effect of miR-101 on cell viability, radiosensitivity and radiation-induced autophagy. (A) Either miR-101 mimic or miRNA-NC was co-transfected into CNE-2 cells with PCA-STMN1 or the empty vector. Western blot analysis was performed to verify the expression of STMN1 48 h following co-transfection. β-Actin was used as an internal control. (B) CNE-2 cells were treated as in A and cell viability was determined with an MTT assay 72 h following co-transfection. (C) Clonogenic survival assays of CNE-2 cells treated as in A followed by various doses of radiation. Surviving fractions were calculated as described. SER10 for miR-101+empty vector, miR-NC+STMN1 and miR-101+STMN1 were 1.42, 0.91 and 1.02, respectively. (D) CNE-2 cells were treated as in A followed by 6 Gy irradiation. Twenty-four hours following IR, western blot analysis was performed to detect the expression of LC3 and p62. β-Actin was used as an internal control. (E) A CNE-2 cell line stably expressing GFP-LC3 was established. These cells were treated as in A followed by 6 Gy IR. Twenty-four hours following IR, cells were fixed in 4% paraformaldehyde and subsequently observed under a confocal microscope (magnification, ×600). The percentage of cells with GFP-LC3 in puncta was calculated in five random fields. Values are presented as the mean ± standard deviation. ^*^P<0.05 vs. empty vector. STMN1, stathmin 1; miR, miRNA; NC, negative control; IR, irradiation.

To determine the functional relevance of STMN1 regulation by miR-101, the effect of STMN1 on miR-101-mediated growth suppression and radiosensitivity was evaluated. STMN1 expression was markedly increased following transfection with PCA-STMN1 plasmid (Fig. 4A). Furthermore, the co-expression of STMN1 markedly rescued the growth suppression and radiosensitivity of CNE-2 cells transfected with the miR-101 mimic (Fig. 4B and C). Additionally, forced expression of STMN1 rescued the downregulation of autophagy-associated protein LC3II and upregulation of p62 protein (Fig. 4D), and decreased the lipidation of LC3 (Fig. 4E), which represented decreased activity of autophagy in the presence of miR-101 24 h following irradiation. These results indicated that STMN1 is one of the key functional targets of miR-101, with respect to the effect of miR-101 on the growth inhibition, radiosensitivity enhancement and autophagy inhibition of NPC cells.

## Discussion

The results of the present study demonstrated that miR-101 was downregulated in NPC cell lines and that IR induced the expression of miR-101. The ectopic expression of miR-101 suppressed the viability of, and enhanced the radiosensitivity of NPC cells. STMN1 was additionally identified as a direct functional target of miR-101 involved in cell growth, radiosensitivity and radiation-induced autophagy. These results suggested that miR-101 has significant roles in the development and radiosensitivity of NPC.

Previous studies have indicated that numerous miRNAs may function as oncogenes, for example miR-155 and miR-21 (22,23), or as tumor suppressors, including the miR-200 family and let-7 (24,25). A loss of miR-101 expression is frequently observed in certain types of human cancer and is associated with therapeutic resistance, which suggests that miR-101 may act as a tumor suppressor (18). In the present study, it was demonstrated that miR-101 was downregulated in NPC cell lines and that the ectopic expression of miR-101 significantly suppressed NPC-cell viability and enhanced their radiosensitivity. This further supported the hypothesized anti-tumor effects of miR-101 in NPC.

A study by Frankel *et al* (7) indicated that miR-101 may act as an inhibitor of autophagy, a catabolic pathway which involves self-degradation and the recycling of macromolecules and cellular organelles. This mechanism has been shown to be a critical adaptive response for tumor cell-survival under stressful conditions, including hypoxia, chemotherapy, radiotherapy or nutrient deprivation, which results in therapeutic resistance (26). Studies have also suggested that blocking autophagy may enhance NPC radiosensitivity (27). In the present study, it was demonstrated that expression of miR-101 was increased in response to IR. This behavior may be an adaptive feedback response to regulate autophagy.

miRNAs mainly regulate their target gene expression via translational repression, mRNA degradation or both (3). Several targets of miR-101, such as histone-lysine *N*-methyltransferase (28) and ATM (29), have been identified. In the present study, a luciferase reporter gene assay verified STMN1 as a direct target of miR-101. In addition, the overexpression of miR-101 significantly reduced the mRNA and protein expression of STMN1. A recent study demonstrated that the expression of STMN1 was upregulated in malignant cancers and correlated with poor prognosis and therapeutic resistance (30). In the present study, it was demonstrated that silencing STMN1 expression was able to suppress NPC-cell proliferation and enhance their radiosensitivity. Furthermore, the forced expression of STMN1 rescued the growth suppression, radiosensitivity and decreased activity of radiation-induced autophagy in NPC cells. This suggested that miR-101 may suppress cell proliferation and enhance radioresistance of NPC cells by directly targeting STMN1. Further investigation is required to elucidate the association between miR-101-mediated autophagy and the radiosensitivity of NPC.

In conclusion, the results of the present study indicated that the downregulation of miR-101 in NPC cell lines and ectopic expression of miR-101 suppressed the cell viability and enhanced the radiosensitivity of NPC cells by directly targeting STMN1. This identified an miR-101/STMN1 pathway which may contribute to the elucidation of the molecular mechanisms by which miR-101 regulates the radiosensitivity of NPC cells. Further investigation will be performed to determine whether miR-101-enhanced radiosensitivity is correlated with the inhibition of autophagy in NPC.
